# Intensity of Mutualism Breakdown Is Determined by Temperature Not Amplification of *Wolbachia* Genes

**DOI:** 10.1371/journal.ppat.1005888

**Published:** 2016-09-23

**Authors:** Chelsie E. Rohrscheib, Francesca D. Frentiu, Emilie Horn, Fiona K. Ritchie, Bruno van Swinderen, Michael W. Weible, Scott L. O’Neill, Jeremy C. Brownlie

**Affiliations:** 1 School of Natural Sciences, Griffith University, Nathan, Australia; 2 Eskitis Institute for Cell and Molecular Therapies, Griffith University, Nathan, Australia; 3 Institute of Health and Biomedical Innovation and School of Biomedical Sciences, Queensland University of Technology, Kelvin Grove, Australia; 4 Queensland Brain Institute, The University of Queensland, St. Lucia, Australia; 5 School of Biological Sciences, Monash University, Clayton, Australia; 6 Environmental Futures Research Institute, Griffith University, Nathan, Australia; Uniformed Services University of the Health Sciences, UNITED STATES

## Abstract

*Wolbachia* are maternally transmitted intracellular bacterial symbionts that infect approximately 40% of all insect species. Though several strains of *Wolbachia* naturally infect *Drosophila melanogaster* and provide resistance against viral pathogens, or provision metabolites during periods of nutritional stress, one virulent strain, *w*MelPop, reduces fly lifespan by half, possibly as a consequence of over-replication. While the mechanisms that allow *w*MelPop to over-replicate are still of debate, a unique tandem repeat locus in the *w*MelPop genome that contains eight genes, referred to as the “Octomom” locus has been identified and is thought to play an important regulatory role. Estimates of Octomom locus copy number correlated increasing copy number to both *Wolbachia* bacterial density and increased pathology. Here we demonstrate that infected fly pathology is not dependent on an increased Octomom copy number, but does strongly correlate with increasing temperature. When measured across developmental time, we also show Octomom copy number to be highly variable across developmental time within a single generation. Using a second pathogenic strain of *Wolbachia*, we further demonstrate reduced insect lifespan can occur independently of a high Octomom locus copy number. Taken together, this data demonstrates that the mechanism/s of *w*MelPop virulence is more complex than has been previously described.

## Author Summary

*Wolbachia* are obligate intracellular, symbiotic bacteria that infect approximately 40% of insect species, as well as filarial nematodes, arachnids and terrestrial isopods. While the vast majority of *Wolbachia* strains impose few fitness costs to their host, one strain *w*MelPop is unique as it lacks the ability to regulate its growth, and as consequence can reduce host lifespan by half. The strength of pathology induced by *w*MelPop has been linked to either increased bacterial density or copy number of an eight gene tandem repeat region referred to as the “Octomom” locus. To date no study has determined the effect changes to temperature have on Octomom copy number or bacterial density. Here we demonstrate that while the Octomom locus is unstable within a single generation of its host, changes to Octomom copy number did not occur in response to temperature. Furthermore, Octomom copy number or bacterial density does not correlate to the strength of pathology. These results indicate that the underpinning genetics of pathology are unclear, and the mechanisms by pathology is induced are more complex than previously realised.

## Introduction

Symbiosis has played a pivotal role in arthropod diversification, speciation, and the ability of these animals to occupy a variety of niches in the natural world. Symbionts fall into two broad categories: infectious symbionts that often impose severe fitness costs to their host in order to complete their lifecycle, and vertically transmitted endosymbionts that form lifelong infections with their host, which range from beneficial to commensal in nature [1]. A key determinate of endosymbiont:host interactions is the infection density that the symbiont establishes in their host [2]. If symbiont density is too high, the endosymbiont risks inducing pathology in the host and reducing host fitness. On the other hand, if density is too low, the endosymbiont may not be transmitted to the next generation [3]. Density of endoysmbionts may be regulated by a combination of microbe or host mechanisms, as well as external factors including nutritional status of the host or temperature [2,4–6].

One symbiont that has been shown to be influenced by all of these factors is *Wolbachia pipientis*, a gram-negative alpha-proteobacteria that infects numerous invertebrate species, such as filarial nematodes and at least 40% of insect species [7–10]. Most *Wolbachia* manipulate host reproductive systems to enhance their maternal transmission through host populations, with a smaller number of strains shown to provide protection against microbial infections [11–13] or impose fitness costs to their host [14–16]. The strength of these phenotypes correlates with *Wolbachia* density [12,17–19], which itself has been shown to be largely strain and host dependent.

Wild populations of *Drosophila melanogaster* are often infected by one of two *Wolbachia* strains, *w*MelCS or more commonly, *w*Mel [20]. A third strain, *w*MelPop, was recovered from a mutant *Drosophila* laboratory stock [15]. The abundance and effect each strain has on *Drosophila* lifespan, or protection against viral infection, correlate with *Wolbachia* density. The *w*Mel strain, which establishes the lowest density in the host, has no impact on lifespan but provides the lowest level of protection against viral infection; conversely *w*MelPop establishes the highest infection density and significantly reduces adult-lifespan but provides the highest level of virus protection [12]. The pathogenicity of *w*MelPop and its ability to over-replicate appear to be independent of host factors, with pathology and associated high infection densities observed in novel transinfected hosts [19], suggesting that genetic factors are responsible. Comparative genomic analyses between *w*MelPop and *w*MelCS have identified an 8-gene region, referred to as the “Octomom” locus, which is triplicated in *w*MelPop [19,21,22]. A recent study by Chrostek and Teixeira also correlated increased copy number of the Octomom locus with both increased *Wolbachia* infection densities and pathology. How the Octomom locus influences pathology was undetermined [22]. A second determinate of *w*MelPop pathology is the extrinsic temperature the host is exposed to, with pathology positively correlating to an increase in temperature [23]. Intriguingly, when flies are reared at 19°C no pathology is observed, presumably because *w*MelPop does not over-replicate or the rate at which it over-replicates was too slow to reduce host fitness [23].

While *w*MelPop pathology has been correlated to increasing temperature [23], or bacterial density and Octomom copy number [22], no studies to date have investigated the effect temperature has on *Wolbachia* density and Octomom copy number. If *Wolbachia* density determines the strength of pathology, we would expect to observe decreasing *Wolbachia* infection densities as the extrinsic temperature decreased. Similarly, if the Octomom copy number determines *Wolbachia* density, and consequently pathology, we would expect to observe a decrease in Octomom copy number as the extrinsic temperature decreases.

Here we evaluated the lifespan of adult Canton-S *Drosophila* infected with *w*MelPop that were reared at four different temperatures, and as expected, observed increased pathology as temperature increased. When flies were reared at 18°C, however, we observed an extension to adult lifespan, similar to that previously observed at 16°C [24]. Estimates of *w*MelPop infection densities showed a bi-modal pattern across the different rearing temperatures, with a distinct shift in bacterial growth in the host. Absolute density and the rate of growth of *Wolbachia* were decoupled from the strength of pathology. Copy number of the Octomom region was dynamic across time but no correlation between temperature and Octomom copy number was observed. Similarly, no correlation between Octomom copy number and bacterial density, or strength of pathology was observed. Finally, we describe a new pathogenic strain of *Wolbachia*, *w*Mel3562, which maintains the Octomom region at low frequency, established a high bacterial infection and reduced adult-lifespan in flies. Taken together, these observations challenge recent evidence of how expansion of the Octomom locus leads to the breakdown of mutualism between *Wolbachia* and host.

## Results

### Survival

**Fig 1:**
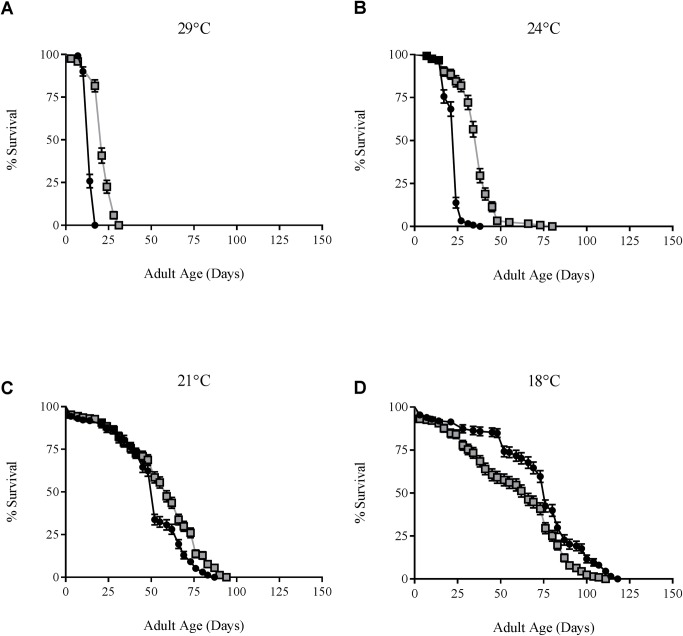
Effect of rearing temperature on survival in flies infected with *w*MelPop. Survival curves of male *w*MelPop-infected flies reared at (A) 29°C, (B) 24°C, (C) 21°C and (D) 18°C. Black-shaded circles represent infected flies, and grey-shaded squares represent *Wolbachia*-uninfected tetracycline treated controls (T). Error bars on curves represent SEs.

A standard survival assay [15] was used to confirm that an increase in environmental temperature correlated with a reduction in adult lifespan of *Drosophila* infected with *w*MelPop. We compared the lifespan of adult *D*. *melanogaster* infected by *w*MelPop across a range of rearing temperatures (29°C, 24°C, 21°C, 18°C), to *Wolbachia*-free controls (Fig 1; Table 1). As expected, we observed the greatest pathology associated with the highest rearing temperature; median survival increased for both infected flies and uninfected controls as rearing temperatures decreased. Additionally, hazard ratios, the ratio of fly death between infected and uninfected controls, decrease with a reduction in temperature (24°C and 21°C), demonstrating that temperature, infection, and their interaction affect *Drosophila* survival (Table 1). When adult *w*MelPop-infected *D*. *melanogaster* were reared at 18°C, *w*MelPop infection was associated with an extended adult lifespan. Similar results have been previously observed for adult flies reared at 16°C [24]. Thus we conclude that there is a temperature dependent effect of *w*MelPop on *Drosophila* survival.

**Table 1:** Survival of *Drosophila melanogaster* infected with *w*MelPop at different rearing temperatures.

Strain, Temperature	N1, N2 (*W* +, *W*-^1^)	df1, df2 (*W* +, *W*-^1^)	Median Survival (Day) (*W* +, *W*-^1^)	Hazard Ratio (95% CI)	*p* Value
***w*MelPop, 29°C**	120, 120	119, 119	14, 21	17.8 (11.7–27.0)	< 0.0001
***w*MelPop, 24°C**	121, 122	120, 121	24, 38	12.2 (8.2–18.1)	< 0.0001
***w*MelPop, 21°C**	231, 234	230, 233	52, 59	1.7 (1.3–2.1)	0.0002
***w*MelPop, 18°C**	198, 203	197, 202	76, 66	0.6 (0.5–0.7)	< 0.0001
***w*Mel3562, 29°C**	206, 199	205, 198	17, 27	11.29 (8.2–15.4)	< 0.0001
***w*Mel3562, 24°C**	198, 214	197, 213	52, 60	1.68 (1.3–2.1)	< 0.0001

1. “*W* -” represent uninfected tetracycline treated paired lines.

### *Wolbachia* density

**Fig 2:**
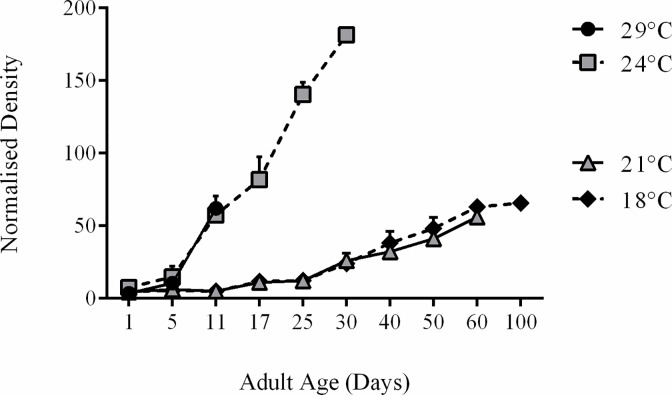
Effect of rearing temperature on *w*MelPop density in *Drosophila melanogaster*. Mean relative *w*MelPop density in flies, as determined by qPCR, reared at four different temperatures. Errors bars on curves represent SEs.

The capacity of a bacterium to cause disease reflects its relative pathogenicity and the degree of virulence is directly influenced by the ability of the organism to cause disease despite host resistance mechanisms; it is affected by different variables such as the number of infecting bacteria [25]. Genes that influence bacteria virulence are also regulated by temperature, which acts as an 'on-off' mechanism for bacterial growth [26]. Using a standard qPCR assay [27], we determined if temperature affected *w*MelPop replication over time in adult *Drosophila*, reared at 29°C, 24°C, 21°C, and 18°C (Fig 2). Flies reared at 29°C and 24°C had equivalent bacterial densities until the last day of fly survival at 29°C (Day 11; *F*(1,8) = 0.59, *p* = 0.61). Over-replication of *w*MelPop continued as flies reared at 24°C aged, reaching an average maximum density of approximately 181.6 *Wolbachia* genomes to 1 *Drosophila* genome. This demonstrated a high bacterial density before death, despite outliving flies at a similar density, reared at 29°C. Flies reared at 24°C and 21°C had significantly different bacterial density until the last day of fly survival at 24°C (*F*(1,8) = 0.02, *p* < 0.0001). Flies reared at 21°C and 18°C had equivalent bacterial densities until the last day of fly survival at 21°C (*F*(1, 8) = 0.64, *p* = 0.22).

*Wolbachia* density displayed a bimodal trend between flies raised at high (29°C and 24°C), and low (21°C and 18°C) temperatures. To determine what temperature would shift *w*MelPop replication from low to high densities, or if an intermediate growth profile existed, we estimated *w*MelPop density in flies reared at 23°C and 22°C. *w*MelPop density in adult *Drosophila* reared at 23°C was not significantly different compared with density in flies reared at 24°C until the last day of fly survival at 24°C (Day 30; *F*(1, 8) = 1.11, *p* = 0.42) a similar result was found when density was measured in flies reared at 22°C was compared to flies reared at 21°C through to the last day of fly survival at 24°C (Day 40; *F*(1,8) = 0.60, *p* = 0.94), demonstrating a bimodal relationship between density and temperature. These results suggest *w*MelPop density is temperature-dependent and that density is not correlated with host pathology under these conditions.

### Octomom copy number

**Fig 3:**
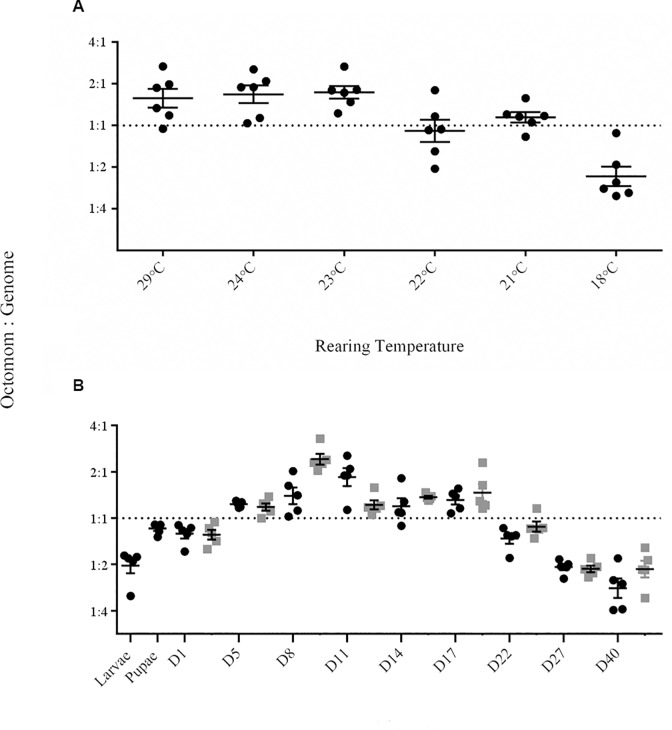
Effect of temperature and fly age on Octomom copy number. (A) Mean Octomom copy number relative to a single copy *w*MelPop gene in 11-day old flies reared at 29°C, 24°C, 23°C, 22°C, 21°C and 18°C, as determined by qPCR. (B) Mean Octomom copy number relative to a single copy *w*MelPop gene in developing flies reared at 24°C (dark-shaded circles) or 21°C (grey-shaded squares) as determined by qPCR. Both flylines were reared at 24°C from embryo to eclosion. Days refer to adult fly age post eclosion. Error bars represent SE.

Chrostek and Teixeira have previously postulated a positive correlation between increasing copy number of the Octomom locus / *w*MelPop bacterial density with pathology [22]. Given that temperature does influence bacterial density, but that bacterial density does not always correlate with pathology, we set out to determine if Octomom copy number correlated with differences observed in *Wolbachia* density at different rearing temperatures, or pathology. Octomom copy number was determined by estimating the ratio of *WD0508*, a single copy gene within the Octomom locus, to *WD0550*, a single copy gene in the *w*MelPop genome. First we compared copy number of *WD0508* in 11-day-old flies, the earliest time point in which the bimodal density trend was observed from six rearing temperatures (29°C, 24°C, 23°C, 22°C, 21°C, and 18°C; Fig 3A). On average, between 1 to 1.5 copies of the Octomom locus were observed in 11-day-old flies reared at 29°C, 24°C, 23°C, 22°C, and 21°C (Fig 3A; S1 Table). These results suggest *Wolbachia* density or pathology is not dependent on Octomom copy in infected *Drosophila*. When *w*MelPop infected flies were reared at 18°C there was a significantly lower prevalence of the insert, with at least half of the bacterial genomes lacking the Octomom locus (Fig 3A). As all flies were maintained at 24°C from embryo to one-day-adult flies prior to being reared at different temperatures, these results suggest either temperature directly affected genome instability at this locus within 11 days or that low temperatures had selected for *w*MelPop variants that lacked or had low copy number of the Octomom locus.

To further explore how stable the Octomom locus was over time, Octomom copy number was estimated in a mixed population of flies reared at 24°C or 21°C, at different time points throughout their lifespan (Fig 3B). If Octomom copy number affected the strength of pathology, flies reared at 21°C should have lower Octomom frequency for a greater period of time when compared to flies reared at 24°C. No significant difference was observed for average Octomom copy number in *w*MelPop infected flies when reared at 24°C and 21°C (Fig 3B; S2 Table). Copy number was initially low in one-day old *w*MelPop infected flies, but increased in frequency by Day 5 and was maintained until Day 17 across both temperatures. Interestingly, as *w*MelPop infected flies aged (Day 22 –Day 40), Octomom copy number was not significantly different from that observed in 11-Day-old flies reared at 18°C (18°C (-2.43 ± 0.46); 24°C (-3.03 ± 0.42), [*F*(1,9) = 1.486 *p* = 0.92]; 21°C (-2.20 ± 0.35), [F(1,9) = 2.06, *p* = 0.59). These results demonstrate that temperature and increasing adult age, not Octomom copy number, influence *w*MelPop bacterial density. Furthermore, we observed Octomom copy number to be highly variable over developmental time and were not correlated to *Wolbachia* density or host pathology.

**Fig 4:**
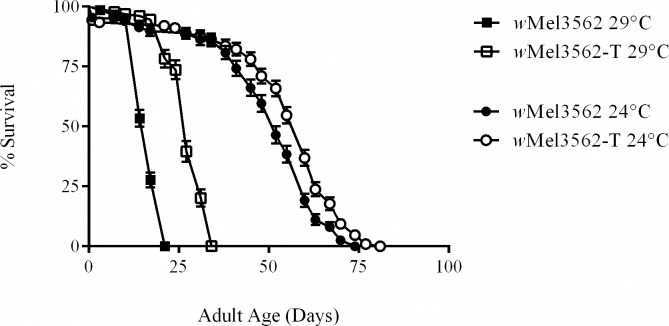
Daily mortality for *w*Mel3562 infected *D. melanogaster*. Survival curves of male *w*Mel3562-infected flies reared at (A) 29°C and (B) 24°C. Black-shaded circles represent infected flies, and grey-shaded squares represent uninfected tetracycline treated controls (T). Error bars on curves represent SE.

**Fig 5:**
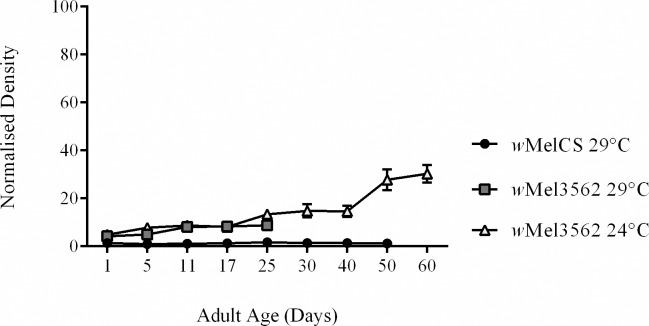
Daily *w*Mel3562 bacterial density in adult *D. melanogaster*. Mean relative *w*Mel3562 density in flies reared at 29°C and 24°C, as determined by qPCR. Errors bars on curves represent SE.

**Fig 6:**
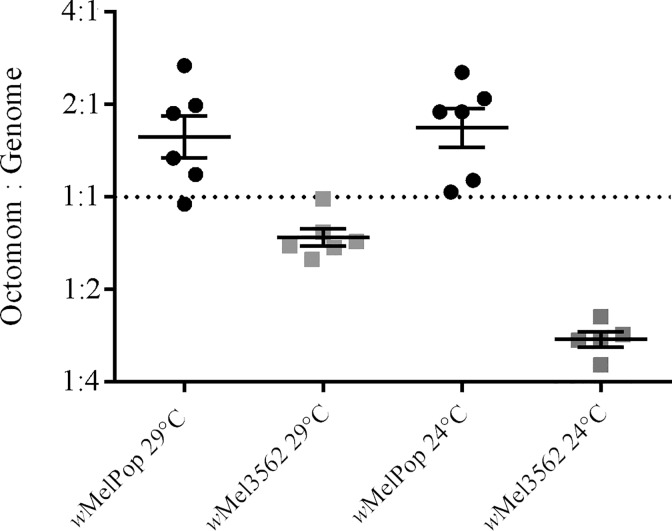
Octomom copy numbers in *w*Mel3562 and *w*MelPop-infected flies.

To date only two *Wolbachia* strains that infect *D*. *melanogaster* are known to establish higher infection densities and reduce adult lifespan: *w*MelCS [12] and *w*MelPop [15] and both were recovered from CantonS *Drosophila* fly-stocks. To determine if other life-shortening *Wolbachia* strains existed we screened short-lived *D*. *melanogaster* fly-stocks for the presence of *Wolbachia* using a standard PCR assay [27]. All *Wolbachia* positive fly-lines were cured of their infection and survival was compared. From fly-stock 3562, a mutagenized CantonS fly-stock which contains a known mutation in the *Hyperkinetic* gene [28], we recovered a pathogenic strain of *Wolbachia* that reduced adult lifespan at both 29°C (Table 1; Fig 4; Hazard Ratio = 11.29; 8.23–15.48) and 24°C (Table 1; Fig 4; Hazard Ratio = 1.68; 1.34–2.11). Genotyping of the strain, hereto referred as *w*Mel3562, confirmed it is a member of the *w*MelCS/*w*MelPop clade sharing all known genetic markers with both strains [12, 13, 20]. We then estimated the *Wolbachia* density (Fig 5) and the copy number of the Octomom locus (Fig 6). Similar to *w*MelPop, *w*Mel3562 over replicated in infected flies (Fig 5) at both 24°C and 29°C, with densities equivalent to those of *w*MelPop observed at 21°C (*F*(1,8) = 2.61; *p* = 0.36), and has a higher density than *w*MelCS when reared at 29°C. As with *w*MelPop pathology, the strength of *w*Mel3562 pathology was influenced by temperature rather than absolute bacterial density or the rate of bacterial growth (Fig 5). Measurements of the Octomom locus in 11-day old flies showed that at 24°C, the frequency of the locus within the *w*Mel3562 population was low (Fig 6; *F*(1,8) = 0.14, *p* < 0.001) compared to *w*MelPop at 24°C, but equivalent to the copy number observed in 22 day-old *w*MelPop infected flies (*F*(1,8) = 0.10, p = 0.86).

Mean Octomom copy number in *w*Mel3562 relative to a single copy *Wolbachia* gene in 11-day old flies reared at 24°C. Errors bars represent SE.

## Discussion

This study provides evidence that *w*MelPop induces host pathology in a temperature dependent manner, which is independent of bacterial density or rate of bacterial growth. Over replication of *w*MelPop was observed in *Drosophila* reared at all evaluated temperatures–the rate at which this was observed was bimodal. Flies reared at high temperatures (29°C—23°C) shared similar bacterial density across time and established the highest bacterial density in the shortest period of time. Flies reared at low temperatures (22°C—18°C) had similar bacterial densities to each other and had a markedly different growth rate and final bacterial density. Interestingly, despite having the same infection density (e.g. 21/18°C or 29/24°C), infected flies lived significantly longer as rearing temperature decreased and even outlived their uninfected counterparts at extremely low temperatures, a phenotype that has been previously described at 19°C and 16°C [23,24]. This suggests that the interaction of *w*MelPop and host rearing conditions is a major determining factor in pathology, and that strength of pathology was not determined by either absolute bacterial density or the rate of growth within the host.

Chrostek and Teixeira recently described a correlation between high and low Octomom copy number, *w*MelPop density and pathology [22]. Critical to these observations were a set of selection experiments whereby they were able to select for high or low-copy Octomom *w*MelPop *Drosophila* flylines and in turn observed altered pathology in accordance with their predictions. A similar selection experiment had been previously conducted, however unlike Chrostek and Teixeira’s experiments, this study concluded that the changes to pathology were due to host effects and not selection acting upon *w*MelPop [29]. The difference between these two studies can be attributed to the design of the selection experiment. While both selected for increased or decreased *w*MelPop pathology for a minimum of 14 generations, based on high/low Octomom copy number [22] or on survival [30], only Carrington and colleague’s experiment comprised a series of backcrosses to the unselected parental stock in order to determine if selection had acted upon the nuclear host genome or the *w*MelPop genome. As the changes to pathology persisted with the host nuclear background they concluded that the observed changes to pathology were due to selection upon the host genome and not the *w*MelPop genome [30]. In the absence of this additional experiment, it is difficult to determine if the selection applied by Chrostek and Teixeira, and its associated changes to pathology, has acted upon *w*MelPop, the host genome or both.

While all estimates of Octomom copy number using qPCR are an average of all *w*MelPop bacteria that infect an individual fly (thus a single fly could harbour both low and high-copy Octomom *w*MelPop bacteria) our data showed that Octomom copy number is highly variable over the fly lifespan. Low copy Octomom *w*MelPop variants were observed in larval, pupal, and late adult insects, and higher Octomom copy *w*MelPop variants present in younger adults. Octomom’s variability over time poses a number of questions and can be explained by a number of scenarios. The first is that low- or high-copy Octomom *w*MelPop variants might be tolerated at different developmental stages, with low-copy number *w*MelPop selected for in larval and pupal stages, while adult flies might tolerate *Wolbachia* with higher copy numbers of Octomom. Variation of Octomom copy number from high- to low-copy number could also be the result of individual flies that harbour high-copy Octomom *w*MelPop dying faster than those infected by low-copy Octomom *w*MelPop. Thus only low-copy Octomom *w*MelPop strains could be recovered in flies older than 17-days. If this were true, then the death rate for flies with low-copy Octomom *w*MelPop should be identical to that of uninfected flies, yet we observed both mortality and *w*MelPop density continue to increase after 17-days of age while at the same time the ratio of the Octomom locus to the rest of the *w*MelPop genome decreased three fold, from a ratio of 1:1 to 1:3 (Fig 3B). A third possibility is that all flies were infected by both low and high-copy Octomom *w*MelPop bacteria, over time the high-copy strains simultaneously induced cellular damage to the host, leading to pathology, and died off faster than low-copy number strains. A final possibility is that due to frequent recombination at the repetitive sequences that flank the Octomom locus, its copy number within the *w*MelPop genome changes over-time as the fly develops and ages.

The role of Octomom copy number variation in pathology is unclear, regardless of how observed variation may arise. We observed no difference in Octomom copy number in *w*MelPop genomes when flies were maintained at different temperatures and harboured different bacterial densities or experienced different levels of pathology. We also showed that in addition to *w*MelPop-CLA [27], the *w*Mel3562 strain establishes an infection density higher than *w*MelCS and induces pathology in the fly host, however, the Octomom region is absent in *w*MelPop-CLA [21] and uncommon within mixed *D*. *melanogaster* populations of *w*Mel3562 (Fig 6). Furthermore, a third strain related to *w*Mel, *w*Au establishes significantly higher infection densities than *w*Mel in *D*. *melanogaster* [30] and also lacks the Octomom region [31,32]. Consequently we conclude that *w*MelPop density, its rate of growth, and the strength of pathology induced is unrelated to the copy number of the Octomom locus.

The mechanisms by which pathogenic strains of *Wolbachia* such as *w*MelPop, *w*MelPop-CLA, and *w*Mel3562 over-replicate and induce pathology as flies age, are still unclear, however temperature appears to be a significant force. It is well established that temperature affects *Drosophila* biology and lifespan. When reared at high temperatures, adult *Drosophila* suffer from a general degeneration of cytoplasmic organelles in nerve cells while at the same time there is an intense loss of ribosomes in the Malpighian tubules [33]. As both tissues are heavily infected by *w*MelPop [15,34–36], the presence of bacteria may exacerbate these physiological responses, leading to the observed host pathology. Temperature has also been shown to affect *Drosophila* immune function and their response to bacterial pathogens [37–39]. When reared at 17°C adult *Drosophila* display increased gene expression of the heat shock protein *Hsp83*, as well as several immune genes, which both correlate with decreased bacterial growth and pathology when compared to flies reared at 25°C or 29°C [38]. Given the decrease in host pathology in *w*MelPop-infected *Drosophila* maintained at low temperatures, it is tempting to speculate that similar host immune responses act to attenuate *w*MelPop pathology.

Despite initial hopes of describing an environment-genotype-to-phenotype link among extrinsic temperature, Octomom copy number and pathology, we have demonstrated that Octomom copy number is highly variable over time, is unresponsive to extrinsic rearing temperature and is not correlated to either bacterial density or pathology. The density of *w*MelPop does not appear to determine pathology as equivalent bacterial densities were observed at different rearing temperatures but the strength of pathology differed. Instead it appears that a combination of the rate of bacterial growth and temperature determines *w*MelPop pathology.

## Methods

### *Drosophila* fly stocks and *Wolbachia* strains

The pathogenic *Wolbachia* strain *w*MelPop [40,41], was introgressed into the *Drosophila melanogaster* Canton-S [42] stocks as described previously [43] *D*. *melanogaster* fly-strain 3562, known to have reduced lifespan and a mapped mutation to *hyperkinetic* (*Hk* ^*1*^) [44,45] was obtained from the Bloomington (Indiana, USA) stock centre. The *Wolbachia* infection from the 3562 flyline, henceforth referred to as *w*Mel3562, was introgressed into two different *Drosophila* genetic backgrounds: BNE, a wild-type strain collected from Brisbane, Australia [46] and *w* ^*1118*^ a heavily inbred white eyed mutant strain. Briefly, virgin 3562 females were mated with *Wolbachia*-free BNE males (BNE-T); female progeny that maintained the *FM6* balancer were collected and crossed to BNE-T males. Wildtype female progeny were collected and backcrossed to the BNE-T background for an additional five generations to create the BNE-*w*Mel3562 line. The *w*Mel3562 strain was introgressed into the *w* ^*1118*^ background from the BNE-*w*Mel3562 flyline by crossing virgin females with *w* ^*1118*^ males for five generations. Tetracycline treatments were performed as described previously [47] to generate genetically identical fly lines that lacked the *Wolbachia* infection; hereto referred as *w*MelPop-T or *w*Mel3562-T. Gut flora was reconstituted using standardised methods [12] and all experiments were conducted at a minimum of seven generations post tetracycline treatment. All flylines were reared from embryo to 1-day-old adults at 24°C on a 12:12 hour light-dark cycle.

### Drosophila lifespan

The lifespan of approximately 200 adult male *Drosophila* derived from *w*MelPop or *w*MelPop-T fly-lines reared as described previously were determined at 29°C, 24°C, 21°C and 18°C. The lifespan of approximately 200 adult male *Drosophila* derived from *w*Mel3562 or *w*Mel3562-T fly-lines reared as described previously were determined at 24°C and 29°C. All adults were collected by CO_2_ anaesthesia immediately following eclosion and separated into groups of 20 before being transferred to the desired temperature, maintained on standard food medium and kept on a 12:12 hour light-dark cycle. Survival was determined every 3-days, until all flies had died. Data was analysed using LogRank analysis (Mantel-Haenszel method; proportional hazards model; SPSS).

### Estimating *w*MelPop density and Octomom insert copy number

Both *Wolbachia* infection density in adult flies and the copy number of the Octomom repeat locus in the *Wolbachia* genome were estimated using relative qPCR assays [27]. Five adult flies reared at 29°C, 24°C, 23°C, 22°C, 21°C, or 18°C were collected at regular time points. Genomic DNA was isolated using a QIAGEN DNeasy Blood & Tissue Kit according to manufacturer instructions (QIAGEN, Doncaster, VIC). Total DNA was estimated using an ND-1000 Nanodrop Spectrophotometer (Analytical Technologies, Collegeville, PA). To estimate the relative abundance of *Wolbachia* bacteria in each sample, we compared the abundance of the single-copy *Wolbachia* ankyrin repeat gene *WD0550* to that of the single-copy *D*. *melanogaster* gene *Act88F* [27]. To estimate the copy number of the Octomom repeat locus, we compared the abundance of the single copy gene *WD0550* to *WD0508* (*WD0508*F: 5’ TGAGGAAGAAAGTGGAAAGGCA 3’ *WD0508*R: 5’ ACATGAGCAGAAACTCCTTCCT 3’), a single copy gene located within the Octomom repeat locus.

Each qPCR contained 12.5 μl of 2x SYBR pre-mix (QIAGEN), 1 μl of Forward primer, 1 μl of Reverse primer, 100 ng of DNA and H_2_O to a final volume of 25 μl. [27]. The relative abundance of *Wolbachia* bacteria to *Drosophila* or Octomom repeat region to the *Wolbachia* genome was determined using the delta-delta CT method [48]. Statistical significance was established with Two-Way ANOVA for bacterial density and One-Way ANOVA for Octomom copy number (SPSS).

## Supporting Information

S1 Table Statistical analysis of Octomom copy number across rearing temperatures (ANOVA). Copy number of WD0508 in 11-day-old flies reared at temperatures (29°C, 24°C, 23°C, 22°C, 21°C, and 18°C).
(DOCX)

S2 Table Statistical analysis of Octomom copy number in flies reared at 24°C and 21°C, across time (2-WAY ANOVA). Copy number of WD0508 in flies reared at 24°C and 21°C across time
(XLSX)

S1 File Raw survival data (*w*MelPop). Raw survival data for flies infected with *w*MelPop.
(XLSX)

S2 File Raw density data. Raw *Wolbachia* density data for flies infected with *w*MelPop or *w*Mel3562.
(XLSX)

S3 File Raw Octomom copy number data. Raw Octomom copy number data for flies infected with *w*MelPop or *w*Mel3562.
(XLSX)

S4 File Raw survival data (*w*Mel3562). Raw survival data for flies infected with *w*Mel3562.
(XLSX)

S5 File Raw Octomom *WD0508* gene expression data. Raw gene expression data for flies infected with *w*MelPop.
(XLSX)
