# Supplementary material for: Intensity of Mutualism Breakdown Is Determined by Temperature Not Amplification of Wolbachia Genes
Source: PLoS Pathog. 2016 Sep 23;12(9):e1005888. doi: 10.1371/journal.ppat.1005888 (PMC5035075; doi:10.1371/journal.ppat.1005888)
Supplement: S1 Table — Copy number of WD0508 in 11-day-old flies reared at temperatures (29°C, 24°C, 23°C, 22°C, 21°C, and 18°C). (DOCX) [file ppat.1005888.s001.docx]

| **Multiple Comparisons** | | | | | | |
| --- | --- | --- | --- | --- | --- | --- |
| Dependent Variable: | Copy_Number |  |  |  |  |  |
| Tukey HSD |  |  |  |  |  |  |
| (I) Temperature | | Mean Difference (I-J) | Std. Error | Sig. | 95% Confidence Interval | |
|  |  |  |  |  | Lower Bound | Upper Bound |
| 29°C | 24°C | -.19500 | .58775 | .999 | -1.9827 | 1.5927 |
|  | 23°C | -.28667 | .58775 | .996 | -2.0744 | 1.5010 |
|  | 22°C | 1.56333 | .58775 | .114 | -.2244 | 3.3510 |
|  | 21°C | .91167 | .58775 | .635 | -.8760 | 2.6994 |
|  | 18°C | 3.73667^*^ | .58775 | .000 | 1.9490 | 5.5244 |
| 24°C | 29°C | .19500 | .58775 | .999 | -1.5927 | 1.9827 |
|  | 23°C | -.09167 | .58775 | 1.000 | -1.8794 | 1.6960 |
|  | 22°C | 1.75833 | .58775 | .056 | -.0294 | 3.5460 |
|  | 21°C | 1.10667 | .58775 | .432 | -.6810 | 2.8944 |
|  | 18°C | 3.93167^*^ | .58775 | .000 | 2.1440 | 5.7194 |
| 23°C | 29°C | .28667 | .58775 | .996 | -1.5010 | 2.0744 |
|  | 24°C | .09167 | .58775 | 1.000 | -1.6960 | 1.8794 |
|  | 22°C | 1.85000^*^ | .58775 | .039 | .0623 | 3.6377 |
|  | 21°C | 1.19833 | .58775 | .345 | -.5894 | 2.9860 |
|  | 18°C | 4.02333^*^ | .58775 | .000 | 2.2356 | 5.8110 |
| 22°C | 29°C | -1.56333 | .58775 | .114 | -3.3510 | .2244 |
|  | 24°C | -1.75833 | .58775 | .056 | -3.5460 | .0294 |
|  | 23°C | -1.85000^*^ | .58775 | .039 | -3.6377 | -.0623 |
|  | 21°C | -.65167 | .58775 | .874 | -2.4394 | 1.1360 |
|  | 18°C | 2.17333^*^ | .58775 | .010 | .3856 | 3.9610 |
| 21°C | 29°C | -.91167 | .58775 | .635 | -2.6994 | .8760 |
|  | 24°C | -1.10667 | .58775 | .432 | -2.8944 | .6810 |
|  | 23°C | -1.19833 | .58775 | .345 | -2.9860 | .5894 |
|  | 22°C | .65167 | .58775 | .874 | -1.1360 | 2.4394 |
|  | 18°C | 2.82500^*^ | .58775 | .001 | 1.0373 | 4.6127 |
| 18°C | 29°C | -3.73667^*^ | .58775 | .000 | -5.5244 | -1.9490 |
|  | 24°C | -3.93167^*^ | .58775 | .000 | -5.7194 | -2.1440 |
|  | 23°C | -4.02333^*^ | .58775 | .000 | -5.8110 | -2.2356 |
|  | 22°C | -2.17333^*^ | .58775 | .010 | -3.9610 | -.3856 |
|  | 21°C | -2.82500^*^ | .58775 | .001 | -4.6127 | -1.0373 |
| *. The mean difference is significant at the 0.05 level. | | | | | | |
